# Latent Profiles of Primary Emotional Systems and Their Associations with PTSD, CPTSD, Attachment, and Childhood Adversity

**DOI:** 10.3390/ejihpe16100162

**Published:** 2026-10-14

**Authors:** Paulo Ferrajão, Rebeca Fernandes Rocha, Amadeu Quelhas Martins

**Affiliations:** 1 Faculdade de Ciências da Saúde, Universidade Europeia, 1500-210 Lisbon, Portugal; rebeca.rocha@ext.universidadeeuropeia.pt (R.F.R.); amadeu.martins@universidadeeuropeia.pt (A.Q.M.); 2 CIDESD—Research Center in Sports Sciences, Health and Human Development, Universidade da Beira Interior, 6200-001 Covilhã, Portugal; 3 UNI-DCOM/IADE, UnidadedeInvestigação em Design e Comunicação, Jardim António Augusto Simenta Mor-dido, Universidade Europeia, 1886-502 Moscavide, Portugal

**Keywords:** affective neuroscience, latent profile analysis, adverse childhood experiences, attachment, posttraumatic stress disorder

## Abstract

Adverse childhood experiences are associated with trauma-related psychopathology, but individual differences in affective organization may contribute to heterogeneous outcomes. This study aimed to identify latent profiles of primary emotional systems and examine their associations with attachment, childhood maltreatment, and posttraumatic stress disorder (PTSD) and complex PTSD (CPTSD). A community sample of 917 Portuguese adults completed measures of primary emotional systems, adult attachment, childhood maltreatment, and PTSD/CPTSD symptoms. Latent profile analyses were conducted separately for women and men, followed by measurement invariance testing and multinomial logistic regression analyses. Four profiles were identified in both sexes: Negative Affect, Low Affective Activation, Balanced Affective Functioning, and Positive Affect. Partial measurement invariance indicated sex-related differences in ANGER, FEAR, and PANIC, while the overall profile structure was comparable across sexes. Negative Affect was associated with PTSD, CPTSD, and fearful attachment in both sexes. Low Affective Activation was associated with CPTSD and dismissing attachment, with additional associations varying by sex. The findings indicate that primary emotional systems form heterogeneous configurations that are differentially associated with developmental, interpersonal, and clinical characteristics. Person-centered assessment may complement symptom-based approaches, although causal inference and profile stability cannot be established.

## 1. Introduction

Exposure to adverse childhood experiences (ACEs) is associated with an increased risk of posttraumatic stress disorder (PTSD), complex posttraumatic stress disorder (CPTSD), and other psychopathological outcomes across the lifespan (Daníelsdóttir et al., 2024). Recent evidence suggests that ACEs affect cognitive, affective, and interpersonal functioning, with emotion dysregulation and maladaptive coping contributing to psychopathological outcomes (Charfi et al., 2026; Guthrie et al., 2026; Khawar et al., 2025). Individual differences in affective and temperamental functioning may therefore contribute to the heterogeneous presentation of trauma-related psychopathology.

In the ICD-11 (World Health Organization [WHO], 2022), PTSD and CPTSD are conceptualized as distinct trauma-related disorders. PTSD is characterized by three core symptom clusters—re-experiencing, avoidance, and a persistent sense of current threat—whereas CPTSD comprises these PTSD symptoms together with disturbances in self-organization (DSO), encompassing affective dysregulation, negative self-concept, and interpersonal difficulties. This distinction suggests that PTSD and CPTSD may involve partially distinct patterns of affective, motivational, and interpersonal dysfunction. Whereas PTSD primarily reflects trauma-related threat responses, CPTSD additionally involves broader disturbances in emotion regulation, self-concept, and interpersonal functioning. Identifying dispositional affective characteristics may therefore help explain variability in posttraumatic outcomes.

Variability in psychological outcomes following ACEs highlights the importance of individual vulnerability and resilience factors in the development of PTSD and CPTSD (Cloitre et al., 2019; Karatzias et al., 2017). Among these factors, temperament and personality are increasingly recognized as important determinants of individual responses to early adversity (Gray & McNaughton, 2000). Childhood trauma is associated with persistent alterations in neurobiological and psychological functioning that may increase vulnerability to psychopathology (Teicher & Samson, 2016). Consistent with this perspective, Affective Neuroscience (AN) theory conceptualizes personality as partly grounded in evolutionarily conserved primary emotional systems that shape affective, motivational, and behavioral responses (Panksepp, 1998; Panksepp & Biven, 2012). These systems may help explain individual differences in trauma-related outcomes.

Primary emotional systems are organized according to their neurobiological functions into positive (approach-oriented) or negative (avoidance-related) systems (Panksepp, 1998). Positive systems include SEEKING, which drives exploration and goal-directed behavior; CARE, which promotes nurturing and affiliative interactions; and PLAY, which supports social bonding and learning through positive social engagement. Negative systems comprise FEAR, which mediates defensive responses to threat; ANGER, activated by frustration or goal obstruction; and PANIC, which underlies distress associated with social separation and loss (Panksepp, 1998; Panksepp & Biven, 2012).

Panksepp’s model has been operationalized through the Affective Neuroscience Personality Scales (ANPS) and its brief version, the Brief Affective Neuroscience Personality Scales (BANPS), which assesses six evolutionarily conserved primary emotional systems: SEEKING, CARE, PLAY, FEAR, ANGER, and PANIC (Davis et al., 2003; Barrett et al., 2013; Panksepp, 1998; Panksepp & Biven, 2012). LUST constitutes an additional primary emotional system in the original model but is generally excluded from brief psychometric operationalizations because of conceptual and methodological limitations in its assessment (Montag et al., 2022).

Within the AN framework, primary emotional systems have been conceptualized as emotional endophenotypes linking biologically based predispositions with observable behavioral and personality phenotypes (Flint & Munafò, 2007; Montag & Davis, 2018). As environmentally sensitive temperamental dimensions, primary emotional systems may contribute to psychopathological vulnerability through individual differences in affective and motivational functioning (Davis & Panksepp, 2018; Montag et al., 2021; Panksepp & Biven, 2012). Importantly, their expression may be shaped by childhood adversity, potentially linking early environmental experiences to enduring affective and behavioral vulnerabilities.

### 1.1. Associations Between Primary Emotional Systems and Attachment

Primary emotional dispositions show evidence of heritability while remaining sensitive to environmental influences, including childhood adversity (Montag et al., 2016). Early adversity has been associated with increased FEAR, ANGER, and PANIC, alongside reduced SEEKING, CARE, and PLAY, reflecting dysregulation of primary emotional systems that may increase vulnerability to anxiety and related psychopathology (Fuchshuber et al., 2019, Fuchshuber & Unterrainer, 2020; Montag & Panksepp, 2017; Watt & Panksepp, 2009). These findings suggest that early adversity may be associated with a broader reorganization of affective functioning rather than with isolated increases in threat sensitivity.

This developmental perspective is supported by evidence linking ACEs to emotion dysregulation and psychopathology. ACEs have been associated with greater emotion dysregulation and symptoms of PTSD, depression, and anxiety, while maladaptive emotion-regulation strategies are linked to greater psychological distress (Feiler et al., 2023; Khawar et al., 2025). Collectively, these findings suggest that early adversity may contribute to enduring alterations in emotional reactivity, regulation, and cognitive-affective functioning (Charfi et al., 2026).

These processes may be particularly relevant to distinguishing PTSD from CPTSD. A recent systematic review and meta-analysis identified emotion regulation, maladaptive coping, attachment, interpersonal relationships, cognitive appraisals, and dissociation as potential pathways linking ACEs to PTSD and CPTSD, with consistent evidence for emotion regulation in the ACEs–PTSD pathway (Guthrie et al., 2026). Recent evidence also highlights the interplay between ACEs, attachment, and emotion regulation (Araújo et al., 2026). Given the limited evidence on mechanisms underlying CPTSD, examining dispositional affective characteristics may help clarify their differential associations with PTSD symptoms and DSO, providing a rationale for examining BANPS profiles rather than trauma-related psychopathology as a unitary outcome.

More specifically, the AN framework provides a basis for expecting partially differentiated associations between primary emotional systems and PTSD versus CPTSD. PTSD may be more closely related to heightened FEAR and, to some extent, ANGER and PANIC, reflecting threat sensitivity and defensive responding (Panksepp, 1998; Panksepp & Biven, 2012). In contrast, the broader affective and interpersonal disturbances characteristic of CPTSD may involve alterations across both negative systems and SEEKING, CARE, and PLAY. Thus, broader dysregulation across negative, approach, and affiliative systems may be particularly relevant to CPTSD, although these propositions remain to be empirically tested.

Attachment theory provides an additional framework for understanding how primary emotional systems may become organized through early interpersonal experience. From an AN perspective, primary emotional systems contribute to attachment and personality through bottom-up processes, while attachment relationships shape higher-order emotional and interpersonal functioning (Davis & Panksepp, 2018; Panksepp & Biven, 2012). In this context, insecure attachment may manifest through attachment anxiety, characterized by heightened concern regarding rejection and hyperactivation of attachment needs, or attachment avoidance, characterized by emotional distancing and suppression of attachment-related needs (Mikulincer & Shaver, 2007).

Within this framework, attachment styles are patterns of cognition, affect, and behavior that regulate interpersonal distress. Secure attachment reflects positive self–other representations and comfort with intimacy, whereas insecure styles include preoccupied (high anxiety and dependence on others for reassurance), dismissive (avoidance and emotional suppression to maintain autonomy), and fearful (anxiety and avoidance, resulting in ambivalent and inconsistent relational patterns) attachment (Mikulincer & Shaver, 2007).

Empirical evidence consistently associates attachment styles with PTSD and CPTSD. Insecure attachment—particularly anxious and fearful—has been positively linked to PTSD symptoms, whereas secure attachment shows an inverse association (Woodhouse et al., 2015). Interpersonal trauma is more strongly related to insecure attachment than non-interpersonal trauma (Barazzone et al., 2019), and fearful and dismissive attachment have been associated with DSO and PTSD symptoms, while secure attachment appears protective (Karatzias et al., 2022).

Primary emotional systems are closely involved in attachment processes, particularly PANIC, which underlies separation distress and proximity-seeking (Panksepp, 2005). Attachment insecurity is associated with elevated PANIC and other negative affective dispositions (Freund et al., 2024; Roithmeier et al., 2024). Secure attachment is characterized by lower ANGER, FEAR, and PANIC and higher SEEKING, PLAY, and CARE, whereas attachment anxiety is linked to increased FEAR, PANIC, and CARE, and attachment avoidance to reduced CARE (Fuchshuber et al., 2019; Roithmeier et al., 2024; Wimmer et al., 2025). Such findings suggest that primary emotional systems and attachment may jointly contribute to trauma-related outcomes.

Taken together, AN, attachment, and emotion-regulation perspectives provide a framework for understanding heterogeneous responses to ACEs (Panksepp & Biven, 2012; Mikulincer & Shaver, 2007). Higher FEAR, ANGER, and PANIC and lower SEEKING, CARE, and PLAY may reflect distinct patterns of threat sensitivity, distress, and reduced approach or affiliative engagement (Davis & Panksepp, 2018; Fuchshuber et al., 2019; Fuchshuber & Unterrainer, 2020). Such configurations may be particularly relevant to CPTSD, given its broader affective, self-related, and interpersonal disturbances. Examining BANPS profiles may therefore help clarify whether PTSD and CPTSD are associated with distinct patterns of primary emotional dispositions.

Previous studies have also reported sex differences in primary emotional systems. Cross-cultural research has shown that females tend to score higher on FEAR and PANIC (Fuchshuber et al., 2019; Sindermann et al., 2017). Higher CARE scores have also been reported among German females, whereas Chinese males exhibited higher PLAY scores (Sindermann et al., 2017). These findings suggest that sex-related differences in primary emotional systems are generally consistent across different cultural contexts, although some domain-specific variations may exist.

### 1.2. Person-Centered Approach to Personality

ANPS research has predominantly employed variable-centered approaches, which assume population homogeneity and examine emotional dimensions independently (Marsh et al., 2009). By contrast, person-centered approaches assume heterogeneity and identify latent subgroups based on configurations of primary emotional systems (Hofmans & Morin, 2024). These approaches enable the identification of empirically derived profiles reflecting qualitative and quantitative differences in affective balance, validated via formal model comparison rather than arbitrary cut-offs (Davis & Panksepp, 2011; Nylund et al., 2007).

Latent profile analysis (LPA) is a person-centered statistical method for identifying unobserved subgroups within a population based on multivariate response patterns (Howard & Hoffman, 2018). Within the ANPS framework, LPA enables the modeling of interactions among primary emotional systems and the identification of distinct affective profiles reflecting both positive and negative emotional dispositions. However, a limited number of studies have applied person-centered approaches to ANPS data.

Orri et al. (2017) were the first to apply LPA to identify affective personality profiles based on ANPS dimensions. Across two independent samples of French-speaking young adults, they identified a three-profile solution. The profiles included a “low negative emotions” group (below-average FEAR and PANIC with average positive affects), a “balanced” profile (average levels across dimensions), and a “high emotional” profile (elevated positive and negative affects). While profile structure was similar across sexes, measurement invariance analyses indicated quantitative differences, with women showing higher CARE, FEAR, and PANIC and lower PLAY.

More recent studies have extended this work to other samples and contexts. Deak et al. (2024) applied LPA to ANPS data in a community sample of 240 adults and identified three profiles reflecting different levels and configurations of primary emotional systems. The profiles included a “high emotional” group (elevated negative emotional systems and a hyperactivation strategy), a “balanced” profile (relatively similar levels of positive and negative emotional systems) and a “low emotional” group (lower positive emotional systems and relatively low negative emotional systems, interpreted in terms of a deactivation strategy). These patterns were broadly replicated in both women and men, although their prevalence differed between sexes.

More recently, Fasolato et al. (2026) conducted a latent profile analysis (LPA) in a clinical sample of patients with eating disorders and identified three distinct profiles. The profiles included a “negative affect dominant” group (high FEAR and PANIC and low PLAY and SEEKING), an “emotionally disengaged” group (low levels of negative emotional systems and CARE), and a “highly emotionally engaged” group (elevated positive emotional systems together with high PANIC and ANGER). These findings support the utility of person-centered approaches for examining the organization of ANPS dimensions across community and clinical samples, while suggesting that both the configuration and prevalence of affective profiles may vary according to sample characteristics and clinical context.

### 1.3. The Present Study

Previous research suggests that ANPS-derived traits capture affective dimensions relevant to trauma-related psychopathology (Brienza et al., 2023; Fuchshuber & Unterrainer, 2020) and are closely related to attachment processes (Davis & Panksepp, 2018; Panksepp & Biven, 2012). Moreover, person-centered studies have consistently identified distinct affective profiles based on ANPS dimensions across community and clinical samples (Orri et al., 2017; Deak et al., 2024; Fasolato et al., 2026), although their specific configuration and prevalence vary across populations. However, to our knowledge, no previous study has examined ANPS-derived affective profiles in relation to PTSD, CPTSD, attachment, and adverse childhood experiences (ACEs) in a community sample.

The present study aimed to identify latent affective profiles based on ANPS dimensions in a Portuguese community sample and to examine their associations with PTSD, CPTSD, attachment styles, and ACEs. Based on the convergence of previous LPA studies, which consistently identified three-profile solutions, we hypothesized that a three-profile solution would adequately capture heterogeneity in the organization of primary emotional systems. Given previous evidence of sex differences in ANPS dimensions and profile prevalence (Fuchshuber et al., 2019; Orri et al., 2017; Sindermann et al., 2017; Deak et al., 2024), we further examined whether profile membership differed between men and women.

Regarding external correlates, we hypothesized that profiles characterized by relatively elevated negative emotional systems and/or reduced positive emotional systems would be associated with greater ACE exposure and a higher likelihood of PTSD and CPTSD, consistent with evidence linking childhood adversity to heightened FEAR, ANGER, and PANIC and reduced SEEKING, CARE, and PLAY (Fuchshuber et al., 2019; Fuchshuber & Unterrainer, 2020; Montag & Panksepp, 2017; Watt & Panksepp, 2009). Conversely, profiles characterized by relatively higher positive emotional systems and lower negative emotionality were expected to show lower ACE exposure and a lower likelihood of PTSD/CPTSD. Finally, we hypothesized that profiles characterized by greater negative emotionality and/or reduced positive emotional systems would be more strongly associated with insecure attachment, particularly fearful and anxious attachment, whereas profiles characterized by relatively higher positive emotional systems would be more strongly associated with secure attachment.

## 2. Materials and Methods

### 2.1. Sample and Study Design

The sample comprised 917 Portuguese adults aged 18–83 years old (M = 35.00, SD = 16.13). Participants were predominantly female, with single individuals representing the largest marital status group, followed by married or cohabiting participants. Most participants had completed secondary education or held a higher education degree. Regarding occupational status, the sample was mainly composed of employed individuals and students, whereas smaller proportions were unemployed or retired. The majority reported a medium socioeconomic status, with fewer participants indicating low, medium–high, or high socioeconomic levels (Table 1).

This study forms part of a broader research project examining the affective dimensions assessed by the BANPS and their associations with psychological functioning and psychopathology in the Portuguese population. Ethical approval was obtained from the Institutional Review Board of Universidade Europeia (ethics approval reference number PI23PF) on 17 July 2024. Participants were recruited through convenience sampling via mailing lists and social media platforms and were required to be Portuguese-speaking adults. Participants were eligible for inclusion if they were aged ≥18 years, reported Portuguese as their first language, and had an educational background sufficient to understand and complete the study questionnaires. Individuals who failed to meet any of these eligibility criteria were excluded from participation. The final sample size was determined by the number of eligible participants who completed the study protocol during the predefined recruitment period (September–December 2024). Given the observational and cross-sectional nature of the study and the use of convenience sampling, recruitment was not terminated on the basis of a prespecified target sample size. Instead, all eligible participants who completed the questionnaire during the recruitment period were retained for analysis. Prior to participation, individuals received detailed information about the study and provided informed consent electronically, in accordance with the ethical principles of the Declaration of Helsinki. Data were collected online using Jotform through a self-report survey assessing primary emotional systems, attachment patterns, adverse childhood experiences, PTSD and CPTSD symptoms, and sociodemographic characteristics. The dataset contained no missing data. This was ensured through the survey design, which required participants to complete all study measures before submitting the questionnaire. Consequently, only fully completed questionnaires were submitted and retained for subsequent analyses. Participation was voluntary and uncompensated, and survey completion required approximately 20 min.

A sensitivity analysis was conducted using the final sample of 917 participants, an alpha level of 0.05, and the generalized linear model framework. The analysis indicated that, under the specified model assumptions, an effect size of η^2^ = 0.01278 corresponded to approximately 80% statistical power. Thus, effects of η^2^ ≥ 0.0168 were expected to be detectable with at least 80% power, whereas smaller effects would have progressively lower power. These results indicate that the study had adequate power to detect effects of small-to-moderate magnitude under the specified model assumptions; however, non-significant findings involving effects smaller than this threshold should be interpreted with caution.

Given the sensitive nature of the questions concerning childhood adversity and PTSD/CPTSD symptoms, participants were informed that participation was voluntary and that they could discontinue the survey at any time without penalty. Participants were also provided with information about appropriate psychological support services in case participation elicited emotional discomfort or distress. Electronic data were stored on encrypted, password-protected systems, with access restricted to authorized members of the research team. The dataset used for statistical analyses was de-identified, and any potentially identifying information was removed prior to analysis. These procedures were implemented to minimize potential risks and ensure the secure handling of sensitive information concerning childhood trauma and mental health.

### 2.2. Measures

The BANPS (Barrett et al., 2013) is a 33-item self-report instrument designed to assess six primary emotional systems underlying affective behavior: ANGER, FEAR, PANIC, PLAY, SEEKING, and CARE. The Portuguese version developed by Gurfinkel et al. (2019) was used in the present study. This version was originally translated and culturally adapted for Brazilian Portuguese. Although the study was conducted in a European Portuguese-speaking sample, this version was selected because it was the Portuguese-language BANPS version available to the research team at the time of data collection. Participants rated their agreement with each item on a five-point Likert scale ranging from 1 (strongly disagree) to 5 (strongly agree), with subscale scores calculated by summing corresponding items. The instrument demonstrated acceptable to good internal consistency, with Cronbach’s α values of 0.81 for ANGER, 0.79 for FEAR, 0.85 for PANIC, 0.83 for PLAY, 0.75 for SEEKING, and 0.78 for CARE.

The Revised Adult Attachment Scale (RAAS; N. L. Collins & Read, 1990; Canavarro, 1997) is an 18-item self-report measure assessing adult attachment across three dimensions—closeness, dependence, and anxiety—using a five-point Likert scale (1 = Not at all characteristic; 5 = Very characteristic). Attachment anxiety was indexed by the anxiety subscale, whereas attachment avoidance was derived from the combined closeness and dependence scores. Categorical attachment styles were defined by participants’ scores on these dimensions: secure (low anxiety, low avoidance), preoccupied (high anxiety, low avoidance), dismissing (low anxiety, high avoidance), and fearful (high anxiety, high avoidance). Specifically, scores above the midpoint of the 5-point response scale were classified as high, whereas scores equal or below the midpoint were classified as low; scores at the midpoint were not assigned to a categorical attachment style. This categorization reflects the original RAAS scoring procedure (N. L. Collins, 1996; N. L. Collins & Read, 1990) rather than a cut-off established specifically for the present study. The scale demonstrated satisfactory internal consistency, with Cronbach’s alpha coefficients of 0.78 for closeness, 0.75 for dependence, and 0.88 for anxiety.

The Childhood Trauma Questionnaire (CTQ; Bernstein et al., 2003; Matos & Pereira, 2012) is a 28-item self-report measure assessing five domains of childhood maltreatment: emotional, physical, and sexual abuse, and emotional and physical neglect. Items are rated on a five-point Likert scale (1—never true to 5—very often true), with higher scores indicating greater trauma severity. Moderate-to-severe exposure was defined using established cut-off scores: emotional abuse (≥13), physical abuse (≥10), sexual abuse (≥8), emotional neglect (≥15), and physical neglect (≥10) (Bernstein & Fink, 1998). In the present sample, the prevalence of moderate-to-severe exposure was 23.1% for emotional abuse, 12.6% for physical abuse, 17.2% for sexual abuse, 20.3% for emotional neglect, and 43.1% for physical neglect. Internal consistency was acceptable to good (Cronbach’s α = 0.86 for Emotional Abuse, 0.84 for Physical Abuse, 0.91 for Sexual Abuse, 0.85 for Emotional Neglect, and 0.73 for Physical Neglect).

The International Trauma Questionnaire (ITQ; Cloitre et al., 2018; Rocha et al., 2022) is an 18-item self-report instrument assessing PTSD and CPTSD in accordance with the ICD-11 diagnostic framework. Items are rated on a five-point Likert scale (0—Not at all to 4—Extremely) and are grouped into six symptom clusters: three assessing core PTSD symptoms (re-experiencing, avoidance, and sense of threat) and three assessing DSO, namely affective dysregulation, negative self-concept, and disturbances in relationships. The occurrence of an index traumatic event was also assessed prior to applying the ITQ diagnostic algorithm; however, this information was not included in the analyses, as examining the characteristics or occurrence of traumatic events was beyond the scope of the present study. PTSD diagnosis requires at least one symptom per PTSD cluster and functional impairment. CPTSD diagnosis requires meeting the PTSD criteria, at least one symptom in each DSO cluster, and functional impairment. Overall, 24.6% of participants met the criteria for PTSD and 10.4% met the criteria for CPTSD. The instrument demonstrated good internal consistency, with Cronbach’s alpha coefficients of 0.88 (PTSD symptoms) and 0.91 (CPTSD symptoms).

Sociodemographic data were collected, including participants’ sex, age, marital status, educational level, occupational status, and socioeconomic background.

### 2.3. Statistical Methods

Latent profile analysis (LPA) was conducted in R (version 4.0) using the tidyLPA package, with standardized mean scores (z-scores) for the six primary emotional systems entered as continuous indicators. Z-scores were calculated using the mean and standard deviation of the total sample rather than separately within sex, thereby preserving potential between-sex differences in the distributions of the indicators for subsequent comparisons across sex. Given established sex differences in primary emotional systems (Orri et al., 2017), separate models were estimated for males and females, evaluating solutions with two to six latent profiles. Models were estimated using a Gaussian finite mixture modeling framework. The models assumed conditional independence of the observed indicators within profiles, with variances constrained to be equal across profiles and covariances fixed to zero, resulting in diagonal variance–covariance matrices. Parameters were estimated using robust maximum likelihood. To reduce the risk of convergence to local optima, a large number of random starting values were employed, with 1000 initial stage starts followed by 250 final-stage optimizations.

Model selection was guided by the integration of statistical fit indices, classification quality, and substantive interpretability. Model fit and parsimony were evaluated using the Akaike Information Criterion (AIC), Bayesian Information Criterion (BIC), and sample-size adjusted Bayesian Information Criterion (SSABIC), with lower values indicating a more favorable balance between model fit and complexity (Lanza et al., 2013). The Bootstrap Likelihood Ratio Test (BLRT) was used to evaluate whether the addition of a latent profile significantly improved model fit relative to the solution with one fewer profile; a non-significant BLRT (*p* > 0.05) was interpreted as supporting the more parsimonious model. Classification quality was examined using entropy, with higher values indicating greater separation among latent profiles. Consistent with previous methodological recommendations, entropy values of approximately 0.40, 0.60, and 0.80 were considered indicative of low, moderate, and high classification precision, respectively. Solutions yielding a smallest profile representing less than 5% of the total sample were interpreted cautiously, with profile prevalence considered in conjunction with statistical fit, classification quality, and theoretical and substantive interpretability (Lo et al., 2001; Masyn, 2013).

Measurement invariance across sex was examined using Multiple-Group Latent Profile Analysis (MGLPA) to assess the comparability of latent profiles between men and women. Following the procedure proposed by L. M. Collins and Lanza (2010), invariance testing proceeded by first evaluating equivalence in the number of profiles across groups. Subsequently, indicator mean invariance was assessed through nested model comparisons between an unconstrained model (M1) and a constrained model with equality constraints across gender (M2), using the likelihood ratio test.

Finally, multinomial logistic regression analyses were conducted to examine the associations between latent profile membership, specified as the dependent variable, and clinical and psychosocial variables, including PTSD and CPTSD diagnoses, attachment styles, and moderate/severe exposure to ACEs, specified as predictor variables. Profile membership was defined according to participants’ most likely latent class, based on their posterior class-assignment probabilities. The Positive Affect profile was specified as the reference category in the multinomial logistic regression model, such that the estimated odds ratios (ORs) represent the odds of membership in each of the remaining profiles relative to this reference profile. For all predictors, statistical significance was evaluated using a two-sided significance level of α = 0.05.

Categorical predictors were entered using binary or dummy coding, as appropriate. For the multinomial logistic regression analyses, each attachment style was operationalized as a binary predictor based on its categorical classification (N. L. Collins, 1996), such that the variable indicated the presence (1) or absence (0) of the respective attachment style. Accordingly, separate binary indicators were created for secure, preoccupied, dismissing, and fearful attachment, with each predictor representing whether participants met the categorical criteria for that specific attachment style. Thus, the regression coefficients and corresponding ORs reflect the association between the presence of a given attachment style and the odds of the outcome, relative to its absence.

For the multinomial regression analyses, CTQ dimensions were categorized according to established cut-off values for the severity of childhood maltreatment, following Bernstein and Fink (1998). For each CTQ domain included in the regression model, categories were recoded into dummy variables, with minimal/low exposure serving as the reference category and moderate/severe exposure as the comparison category. Thus, the corresponding ORs represent the relative odds of membership in each latent profile for participants reporting moderate/severe levels of the respective form of childhood maltreatment compared with those reporting minimal/low levels. This procedure was applied to the relevant CTQ domains, including emotional abuse, physical abuse, sexual abuse, emotional neglect, and physical neglect.

For the diagnostic analyses, PTSD and CPTSD diagnoses were established using the International Trauma Questionnaire (ITQ; Cloitre et al., 2018; Rocha et al., 2022), in accordance with the ICD-11 diagnostic framework. The ITQ comprises 18 self-report items assessing the core symptom clusters of PTSD—re-experiencing, avoidance, and sense of threat—and the three domains of disturbances in self-organization (DSO), namely affective dysregulation, negative self-concept, and disturbances in relationships. Diagnostic status was determined according to the established ITQ diagnostic algorithm. PTSD was considered present when participants endorsed at least one symptom within each of the three PTSD clusters and reported associated functional impairment. CPTSD was considered present when participants met the diagnostic criteria for PTSD, endorsed at least one symptom within each of the three DSO clusters, and reported functional impairment. Based on these criteria, participants were classified into three mutually exclusive diagnostic categories: no PTSD/CPTSD, PTSD, or CPTSD. For the regression analyses, these categories were dummy-coded, with participants who did not meet diagnostic criteria for either PTSD or CPTSD serving as the reference category. Thus, the corresponding ORs represent the relative odds of membership in each latent profile for participants meeting diagnostic criteria for PTSD or CPTSD compared with those who did not meet criteria for either disorder.

Although all constructs were assessed using a single online self-report survey, several procedural safeguards were implemented to reduce the potential for common method variance (CMV), including the use of established and psychometrically validated measures, clear and standardized instructions, anonymous participation, and the separation of conceptually distinct constructs within the survey. In addition, consistent with recommendations for assessing CMV, a marker-variable analysis was conducted using Protection of Nature as the marker variable. This variable was selected because it has limited theoretical overlap with the substantive constructs examined in the present study. Pearson correlations between the marker variable and all substantive study variables were examined to assess the extent to which the marker variable shared variance with the measures used in the study.

## 3. Results

### 3.1. Latent Profile Analysis

#### 3.1.1. Latent Profile Analysis of the Female Subgroup

In the female subgroup, the four-profile solution was identified as providing an optimal representation of the data, achieving an appropriate balance between goodness of fit and model parsimony (see Table 2 for comparative model indices). Although the AIC and SSABIC continued to decrease for the five- and six-class solutions, the increase in BIC beyond the four-class model suggested that additional classes did not improve model fit when model complexity was taken into account. Furthermore, the four-class solution yielded the highest entropy, indicating superior classification accuracy while maintaining adequately large class sizes. Collectively, these findings support the four-class solution as offering the most appropriate balance among model fit, parsimony, and interpretability.

**Figure 1:**
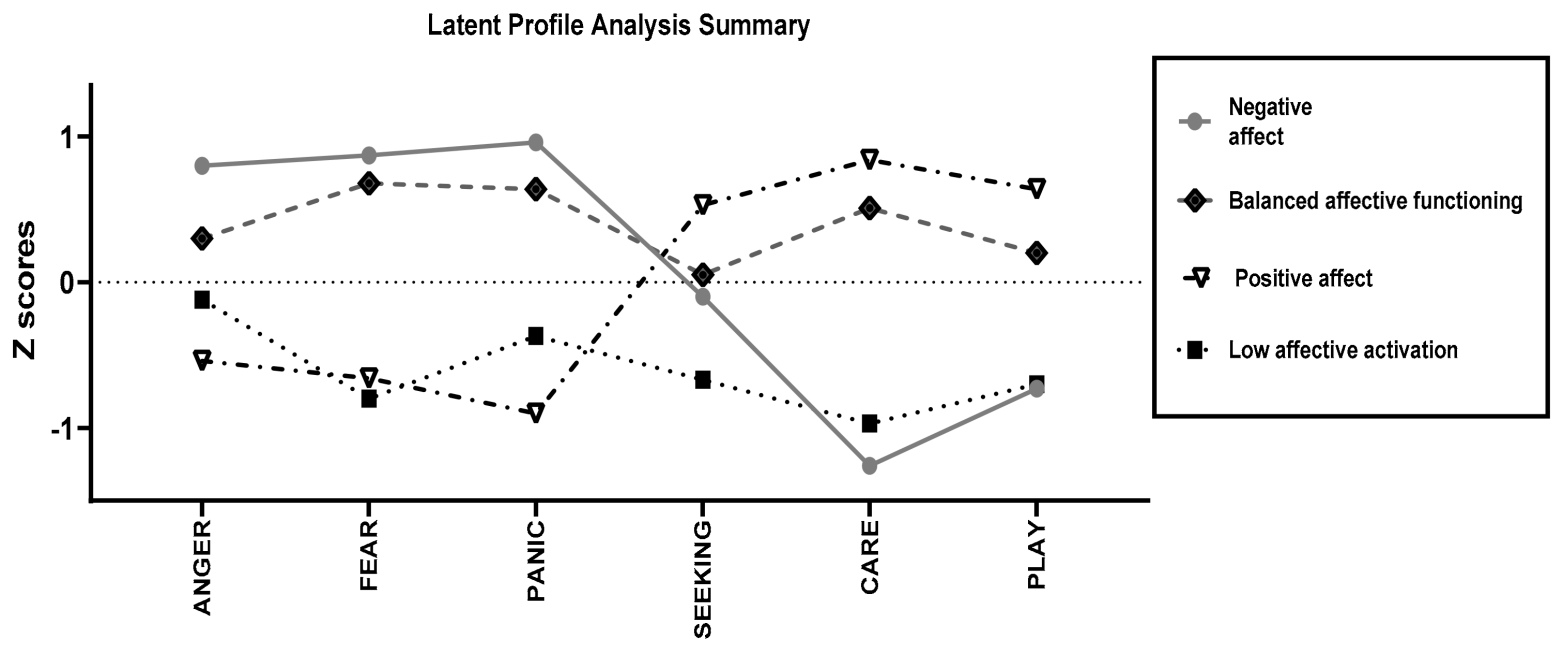
Latent profiles of primary emotional systems in the female subgroup.

Figure 1 illustrates the standardized mean estimates of the indicator scales across the four identified profiles.

Profile 1 was the smallest group, comprising 89 participants (15.4%). It was characterized by markedly elevated levels of ANGER, FEAR, and PANIC (all substantially above the sample mean), together with markedly reduced levels of CARE and PLAY and near-average levels of SEEKING. This pattern suggests a predominance of negative emotional reactivity accompanied by diminished prosocial and exploratory tendencies. Accordingly, this profile was labeled the “negative affect” profile.

Profile 2 comprised 101 participants (17.4%) and was characterized by consistently below-average scores across all six dimensions, with particularly low levels of CARE and SEEKING. ANGER levels were slightly below the sample mean, whereas FEAR and PANIC levels were also below the sample mean. This pattern was characterized by a generalized attenuation across emotional dimensions, suggesting relatively low affective activation in both negative and positive domains. Accordingly, this profile was designated the “low affective activation” profile.

Profile 3 was the largest group, comprising 269 participants (46.5%), and was characterized by moderately above-average scores in FEAR, PANIC, CARE, and PLAY, with ANGER slightly above and SEEKING close to the sample mean. This pattern suggests a relatively balanced configuration of affective systems, characterized by moderate activation of both negative and positive emotional dimensions. Accordingly, this profile was designated the balanced “affective functioning” profile.

Finally, Profile 4 comprised 120 participants (20.7%) and was characterized by below-average scores in ANGER, FEAR, and PANIC, alongside elevated levels of SEEKING, CARE, and PLAY. This pattern reflects low levels of negative emotionality coupled with heightened activation of positive, affiliative, and exploratory systems. Accordingly, this profile was labeled the “positive affect” profile.

To further assess classification quality, average posterior probabilities were examined for each latent profile. The average posterior probabilities were 0.800 for the Negative Affect profile, 0.844 for the Low Affective Activation profile, 0.861 for the Balanced Affective Functioning profile, and 0.879 for the Positive Affect profile. These values indicate a generally adequate level of classification certainty across profiles, although classification was comparatively less certain for the Negative Affect profile.

#### 3.1.2. Latent Profile Analysis of the Male Subgroup

Consistent with the findings observed in the female subgroup, the four-profile solution provided the most parsimonious and substantively interpretable representation of the data in the male subgroup (see Table 3). Although the AIC and SSABIC continued to decrease and the BLRT remained statistically significant with the addition of further profiles, these improvements in statistical fit were accompanied by increasing model complexity. Notably, the BIC increased when moving from the four- to the five-profile solution, suggesting that the additional profile did not provide sufficient improvement in model fit to offset the increased complexity of the model. Although entropy values increased across successive solutions, indicating progressively greater classification precision, this improvement was accompanied by the emergence of increasingly small profiles. Accordingly, the four-profile solution was retained based on the joint consideration of statistical fit, parsimony, classification quality, profile size, and substantive interpretability. This solution therefore provided the most balanced representation of the underlying heterogeneity in the male subgroup without introducing additional profiles that offered limited substantive benefit relative to their increased complexity.

**Figure 2:**
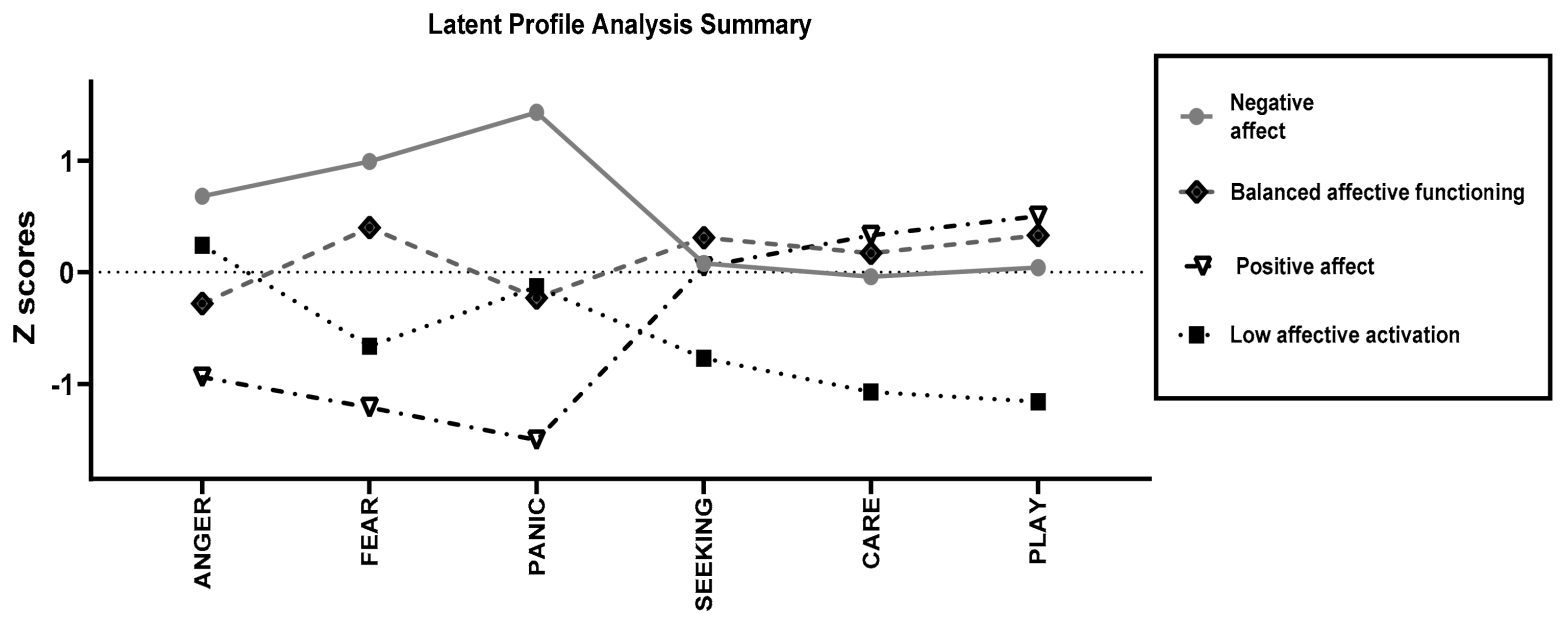
Latent profiles of primary emotional systems in the male subgroup.

Figure 2 displays the profile-specific standardized mean estimates (z-scores) of the indicator scales across the four identified profiles.

Consistent with the pattern identified in the female subgroup, Profile 1 constituted the smallest group, encompassing 33 participants (9.8%). Participants in this profile exhibited above-average levels of ANGER, FEAR, and especially PANIC, suggesting a pronounced activation of negative affective systems. Conversely, SEEKING, CARE, and PLAY scores were comparable to the sample mean, indicating the preservation of approach-related and affiliative dispositions, albeit at moderate levels. Accordingly, this group was labeled the “negative affect” profile.

Profile 2 comprised 73 participants (21.6%) and was characterized by predominantly below-average scores across most affective dimensions, with particularly low levels of FEAR, SEEKING, CARE, and PLAY. As observed in the female subgroup, ANGER was the only dimension to score slightly above the sample mean. This configuration suggests a broadly diminished activation of primary emotional systems, encompassing both aversive and appetitive domains, albeit with some degree of heterogeneity across dimensions. Accordingly, this profile was designated the “low affective activation” profile.

Profile 3 was the largest group, comprising 156 participants (46.2%), and was characterized by scores clustering around the sample mean across all six affective dimensions, with only minor deviations, namely slightly lower PANIC and slightly higher PLAY scores. This pattern indicates a relatively balanced activation of both negative (ANGER, FEAR, and PANIC) and positive (SEEKING, CARE, and PLAY) emotional systems, without marked elevations or reductions in any specific domain. Similar to the pattern identified in the female subgroup, this profile appears to reflect a normative level of affective functioning. Accordingly, it was designated the “balanced affective functioning” profile.

Finally, Profile 4 comprised 76 participants (22.5%) and was characterized by markedly below-average scores in ANGER, FEAR, and particularly PANIC, indicating minimal activation of distress-related affective systems. In contrast, SEEKING, CARE, and PLAY scores were above the sample mean, with PLAY exhibiting the highest elevation. As observed in the female subgroup, this pattern is indicative of enhanced activation of positive emotional systems coupled with reduced activation of negative affective systems. Accordingly, this profile was designated the “positive affect” profile.

To further assess classification quality, average posterior probabilities were examined for each latent profile. The average posterior probabilities were 0.863 for the Negative Affect profile, 0.877 for the Low Affective Activation profile, 0.838 for the Balanced Affective Functioning profile, and 0.852 for the Positive Affect profile, indicating generally high classification certainty across the four profiles.

### 3.2. Measurement Invariance Across Sexes

Measurement invariance across sex was examined using MGLPA, with the results presented in Table 4. Comparison of the unconstrained model (M1) with the fully constrained model (M2), in which all profile-specific indicator means were constrained to equality across males and females, revealed a significant decrement in model fit (ΔLL = 186, χ^2^(21) = 372, *p* < 0.001). This finding indicates that full measurement invariance across sex was not supported.

Given that full measurement invariance across sex was not established, subsequent analyses focused on examining sex-related differences in the six primary emotional systems using multivariate analysis of variance (MANOVA). Statistically significant differences were identified among the profiles on the combined dependent variables, F(6, 910) = 14.56, *p* < 0.001, η^2^ = 0.09. Bonferroni-adjusted comparisons were used to control multiple testing. Significant effects of sex were observed for ANGER, FEAR, and PANIC, with females reporting higher scores than males. No significant sex differences emerged for the remaining emotional systems (Table 5).

Given the significant sex differences identified for ANGER, FEAR, and PANIC in the subsequent MANOVA, a partially constrained model (M2a) was estimated in which the profile-specific means of these three emotional systems were freely estimated across males and females, while the remaining indicator means were constrained to equality. As reported in Table 4, the partially constrained model yielded a lower BIC (ΔBIC = −346) and slightly higher entropy (ΔEntropy = 0.018) relative to the unconstrained model, providing support for a partial invariance specification in which sex-related differences were confined to ANGER, FEAR, and PANIC.

Overall, the findings support a partially invariant latent profile solution across sexes rather than full measurement invariance. Although the latent profile structure was retained across males and females, equality constraints on all profile-specific indicator means were not supported, with sex-specific variation primarily identified for PANIC, CARE, and FEAR. Consistent with these findings, the MANOVA indicated significant sex differences in ANGER, FEAR, and PANIC. Accordingly, subsequent analyses should consider sex-specific parameter estimates for indicators showing evidence of meaningful between-sex differences.

### 3.3. Multinomial Logistic Regression

Multinomial logistic regression analyses were conducted separately for males and females due to the lack of measurement invariance across sex, with most likely profile membership as the dependent variable. Predictors included PTSD and CPTSD diagnoses, attachment styles, and moderate/severe exposure to the five ACEs dimensions, all coded as dichotomous variables (yes or no).

#### 3.3.1. Female Subgroup

Table 6 shows the odds ratio tests for the multinomial logistic regression for the female subgroup.

Regarding moderate-to-severe exposure to specific ACEs, emotional abuse, physical abuse, and emotional neglect were significant predictors of group membership. Compared with the reference group, participants in the Negative Affect group had approximately twice the odds of reporting moderate-to-severe emotional neglect. Participants in the Low Affective Activation group had nearly five times the odds of reporting moderate-to-severe physical abuse, whereas those in the Balanced Affective Functioning group had more than 2.5 times the odds of reporting moderate-to-severe emotional abuse.

Regarding attachment styles, secure, fearful, dismissing, and preoccupied attachment were significant predictors of profile membership, with distinct patterns of association across profiles. Compared with the reference profile, participants in the Negative Affect profile had approximately 5.5 times the odds of reporting fearful attachment, whereas secure attachment was associated with 77% lower odds of membership in the Negative Affect profile. Membership in the Low Affective Activation profile was associated with nearly three times the odds of reporting dismissing attachment, while participants in the Balanced Affective Functioning profile had approximately four times the odds of reporting preoccupied attachment.

Both PTSD and CPTSD diagnoses were significantly associated with profile membership. Compared with the reference profile, participants in the Negative Affect profile had approximately 2.5 times the odds of meeting the criteria for PTSD, while those in the Balanced Affective Functioning profile had slightly more than twice the odds. For CPTSD, both the Negative Affect and Low Affective Activation profiles were associated with increased odds of meeting diagnostic criteria, with each showing more than twice the odds compared with the reference profile.

#### 3.3.2. Male Subgroup

Table 7 presents the odds ratios derived from the multinomial logistic regression analysis conducted for the male subgroup.

Regarding ACEs, moderate-to-severe emotional abuse, physical abuse, emotional neglect, and physical neglect were significant predictors of profile membership. Compared with the reference profile, participants in the Negative Affect profile had more than 2.5 times the odds of reporting moderate-to-severe emotional abuse and approximately 2.5 times the odds of reporting moderate-to-severe physical neglect. Membership in the Low Affective Activation profile was associated with more than 5.5 times the odds of reporting moderate-to-severe physical abuse and five times the odds of reporting moderate-to-severe emotional neglect.

Regarding attachment styles, secure, dismissing, and fearful attachment significantly predicted profile membership. Compared with the reference profile, participants in the Negative Affect profile had six times the odds of reporting fearful attachment, while secure attachment was associated with 80% lower odds of membership in this profile. In the Low Affective Activation profile, participants had nearly twice the odds of reporting dismissing attachment and more than twice the odds of reporting fearful attachment, whereas secure attachment was associated with 68% lower odds of membership in this profile.

Regarding trauma-related psychopathology, PTSD and CPTSD diagnoses were significant predictors of profile membership, particularly in the Negative Affect and Low Affective Activation profiles. Compared with the reference profile, participants in the Negative Affect profile had nearly three times the odds of meeting the criteria for PTSD and more than 3.5 times the odds of meeting the criteria for CPTSD. Participants in the Low Affective Activation profile had more than 2.5 times the odds of meeting the criteria for CPTSD.

### 3.4. Assessment of Common Method Variance

To further assess the potential influence of common method variance (CMV), a marker-variable analysis was conducted, following the reviewer’s recommendation. Protection of Nature was selected as the marker variable because it was not theoretically expected to overlap substantially with the substantive constructs examined in the present study. The marker variable was correlated with all variables included in the analyses to assess the extent to which it shared variance with the substantive measures.

The correlations between Protection of Nature and the six primary emotional systems were small, ranging from r = −0.09 to r = 0.13 (ANGER, r = −0.09, *p* = 0.01; FEAR, r = −0.01, *p* = 0.81; PANIC, r = −0.08, *p* = 0.02; SEEK, r = 0.13, *p* = 0.01; CARE, r = 0.12, *p* = 0.01; PLAY, r = 0.08, *p* = 0.02). Correlations with CLOSENESS (r = −0.12, *p* = 0.01), DEPENDENCE (r = 0.03, *p* = 0.32), and ANXIETY (r = −0.04, *p* = 0.25) were also small. Similarly, correlations with emotional abuse (r = −0.01, *p* = 0.89), physical abuse (r = 0.02, *p* = 0.50), sexual abuse (r = −0.02, *p* = 0.46), emotional neglect (r = −0.02, *p* = 0.54), and physical neglect (r = −0.02, *p* = 0.66) were negligible. Finally, correlations with PTSD global score (r = −0.01, *p* = 0.82) and CPTSD global score (r = 0.03, *p* = 0.42) were negligible. Overall, all correlations between the marker variable and the substantive variables were weak, with absolute correlations not exceeding 0.13. These findings provide no indication of a substantial common method effect and suggest that CMV is unlikely to account for a substantial proportion of the observed associations. Nevertheless, given that all measures were collected using self-report questionnaires within a single survey, the possibility of some residual common method influence cannot be completely excluded and should be considered when interpreting the findings.

## 4. Discussion

This study extends the limited evidence on the role of primary emotional systems in trauma-related psychopathology by identifying distinct affective profiles and examining their associations with PTSD and CPTSD, attachment styles, and ACEs. Consistent with previous latent profile research (Deak et al., 2024; Fasolato et al., 2026; Orri et al., 2017), the findings indicate substantial heterogeneity in the configuration of primary emotional systems and suggest that these affective patterns are meaningfully related to developmental and psychopathological characteristics. By integrating AN and attachment theory, this study contributes to a more nuanced understanding of the heterogeneous affective patterns associated with PTSD and CPTSD. Importantly, given the cross-sectional and self-report nature of the data, these associations should be interpreted as correlational and theoretically consistent with, rather than demonstrative of, specific developmental or causal pathways.

### 4.1. Profiles of Primary Emotional Systems

The identification of four distinct profiles of primary emotional systems in both women and men further supports the conceptualization of affective personality within the Affective Neuroscience framework as a multidimensional and internally differentiated construct. Although a three-profile solution was hypothesized based on previous person-centered studies (Orri et al., 2017; Deak et al., 2024; Fasolato et al., 2026), the present findings supported a more differentiated structure. This suggests that primary emotional systems may combine into distinct affective configurations within individuals, extending previous work by highlighting the heterogeneity in their organization at the individual level.

The four profiles showed both continuity with and differences from previous LPA findings. The Low Affective Activation profile shared features with the “low emotional” and “emotionally disengaged” profiles identified by Deak et al. (2024) and Fasolato et al. (2026), respectively, whereas the Negative Affect and Positive Affect profiles reflected more clearly differentiated negative- and positive-affective configurations. The Balanced Affective Functioning profile was consistent with the balanced profiles reported previously (Orri et al., 2017; Deak et al., 2024). Overall, these findings suggest that broadly similar affective configurations may emerge across studies, while their specific differentiation and prevalence may vary across samples and contexts.

The Negative Affect profile was characterized by elevated ANGER, FEAR, and PANIC, reflecting greater expression of primary emotional systems involved in frustration, threat, and social separation, respectively (Panksepp, 1998; Panksepp & Biven, 2012). This configuration is broadly consistent with previous evidence linking heightened negative primary emotional systems to emotional dysregulation and greater psychopathological vulnerability (Brienza et al., 2023; Fuchshuber et al., 2019). Overall, the profile reflects a predominance of negative affective systems relative to the more balanced or positive configurations identified in the present study, although the cross-sectional design does not allow conclusions regarding the mechanisms underlying this pattern.

Sex-related differences within this profile indicated that similar levels of negative affective expression may coexist with different configurations of positive emotional systems. Women showed lower CARE and PLAY alongside elevated negative systems, whereas men showed comparatively average levels of positive systems. This pattern is broadly consistent with previous evidence of higher FEAR and PANIC among women and sex-related variation in CARE and PLAY (Fuchshuber et al., 2019; Sindermann et al., 2017). Rather than indicating fundamentally different affective structures, these findings suggest quantitative differences in the expression of specific primary emotional systems within a broadly comparable profile.

The Low Affective Activation profile was characterized by comparatively low levels across both positive and negative primary emotional systems. This configuration shares important features with the “low emotional” profile identified by Deak et al. (2024) and the “emotionally disengaged” profile reported by Fasolato et al. (2026), although the specific patterns differed across studies. Notably, lower levels of negative systems co-occurred with reduced SEEKING, CARE, and PLAY, suggesting a broader pattern of diminished affective activation rather than simply low negative emotionality. Although this configuration may be compatible with reduced affective engagement, the present measures did not directly assess emotional numbing, dissociation, anhedonia, or interpersonal withdrawal. Accordingly, the profile should be interpreted as a pattern of relatively low expression across the assessed primary emotional systems rather than as a direct indicator of these processes.

The similarity of this profile across women and men further suggests that low affective activation was not strongly differentiated by sex in the present sample. More broadly, its identification highlights an aspect of affective organization that may be overlooked when positive and negative systems are considered separately. A person may report relatively low levels of negative emotionality while simultaneously showing reduced expression of motivational, affiliative, and social-engagement systems. This distinction is consistent with the conceptualization of SEEKING, CARE, and PLAY as systems involved in exploration, affiliation, and positive social engagement (Brienza et al., 2023; Panksepp, 1998; Panksepp & Biven, 2012), while remaining agnostic about the mechanisms underlying their co-occurrence.

The Balanced Affective Functioning profile was characterized by relatively moderate levels across the primary emotional systems, broadly resembling the balanced profiles identified by Orri et al. (2017) and Deak et al. (2024). This convergence suggests some consistency in the identification of an intermediate affective configuration across community samples. However, this profile should not be considered inherently optimal or adaptive, but rather as reflecting a relatively balanced organization of positive and negative emotional systems. Women showed higher FEAR, PANIC, CARE, and PLAY than men, consistent with previous evidence of sex-related variation in primary emotional systems (Fuchshuber et al., 2019; Sindermann et al., 2017).

Finally, the Positive Affect profile was characterized by elevated SEEKING, CARE, and PLAY alongside lower ANGER, FEAR, and PANIC, reflecting a more distinctly positive-affective configuration (Panksepp, 1998; Panksepp & Biven, 2012). Unlike the “high emotional” and “highly emotionally engaged” profiles reported by Orri et al. (2017) and Fasolato et al. (2026), respectively, which combined elevated positive and negative systems, this profile showed a clearer predominance of positive systems. Sex-related differences were also observed, with men showing higher PLAY and lower PANIC, whereas women showed higher CARE, consistent with previous findings (Fuchshuber et al., 2019; Sindermann et al., 2017).

Measurement invariance analyses indicated that full invariance across sex was not supported, as constraining all profile-specific means to equality resulted in a significant reduction in model fit. Subsequent analyses identified higher ANGER, FEAR, and PANIC scores among females, whereas the remaining emotional systems did not differ significantly by sex. A partially constrained model allowing sex-specific estimates for these three systems provided improved fit, supporting partial measurement invariance. This pattern is broadly consistent with previous ANPS person-centered research showing comparable affective configurations across sex alongside quantitative differences in specific emotional systems, particularly FEAR and PANIC (Orri et al., 2017; Deak et al., 2024). These findings highlight the utility of person-centered approaches for characterizing the heterogeneous co-occurrence of primary emotional systems, while underscoring the importance of accounting for sex-related differences when interpreting ANPS profiles. Nevertheless, the cross-sectional and self-report design precludes conclusions regarding developmental or causal pathways or the proposed neurobiological basis of these profiles.

### 4.2. Primary Emotional Profiles as Markers of Vulnerability and Resilience

Multinomial logistic regression analyses revealed systematic associations between affective profile membership, ACEs, attachment styles, and trauma-related psychopathology. Overall, these findings indicate that distinct configurations of primary emotional systems were differentially associated with developmental and clinical characteristics, consistent with person-centered and AN perspectives (Hofmans & Morin, 2024; Panksepp & Biven, 2012). However, given the cross-sectional design, these associations should not be interpreted as evidence of developmental or causal pathways.

The Negative Affect profile showed consistent associations with trauma-related psychopathology and fearful attachment across both sexes. In women, membership in this profile was associated with higher odds of PTSD and CPTSD and with substantially greater odds of fearful attachment, while secure attachment was inversely associated with profile membership. A comparable pattern emerged among men, with the Negative Affect profile associated with PTSD, CPTSD, and fearful attachment, as well as with lower odds of secure attachment. These findings are consistent with the conceptualization of FEAR and PANIC as affective systems involved in threat sensitivity and separation-related distress (Montag & Panksepp, 2017; Panksepp, 2005). They also agree with previous evidence linking insecure, particularly fearful, attachment with trauma-related psychopathology (Barazzone et al., 2019; Karatzias et al., 2022). These findings suggest that a profile characterized by relatively greater negative affective activation co-occurred with interpersonal insecurity and trauma-related psychopathology in both sexes.

Associations between ACEs and the Negative Affect profile differed by sex. Among women, emotional neglect was associated with membership in this profile, whereas among men, emotional abuse and physical neglect were associated with profile membership. These findings are broadly consistent with evidence linking childhood adversity to alterations in affective functioning, emotion regulation, and threat-related processes (Fuchshuber et al., 2019; Teicher & Samson, 2016). However, the sex-specific pattern observed in the present study should be interpreted cautiously, as differences in the types of adversity associated with profile membership do not by themselves establish distinct developmental mechanisms.

The Low Affective Activation profile was particularly associated with CPTSD across sexes. In women, this profile was also associated with severe physical abuse and dismissing attachment, whereas in men it was associated with severe physical abuse, emotional neglect, dismissing attachment, and fearful attachment. These findings may be relevant to the broader affective and interpersonal disturbances characteristic of CPTSD, including difficulties in emotion regulation and relationships (Cloitre et al., 2019; Karatzias et al., 2017). The association with dismissing attachment across sexes is also consistent with theoretical accounts linking attachment avoidance to emotional distancing and reduced engagement with attachment-related needs (Mikulincer & Shaver, 2007). Nevertheless, the present findings do not establish whether lower affective activation represents a pre-existing vulnerability, a correlate, or a consequence of trauma-related psychopathology.

The Balanced Affective Functioning profile showed a more specific pattern of associations among women. Membership in this profile was associated with emotional abuse, preoccupied attachment, and PTSD, whereas these associations were not observed among men. The association with preoccupied attachment is compatible with previous evidence linking attachment anxiety with heightened FEAR, PANIC, and CARE-related processes (Fuchshuber et al., 2019; Mikulincer & Shaver, 2007; Roithmeier et al., 2024). The concurrent association with PTSD suggests that relatively balanced levels of primary emotional systems do not necessarily imply the absence of trauma-related psychopathology. Rather, the findings indicate that similar overall affective configurations may co-occur with different developmental and clinical characteristics depending on sex.

Taken together, the sex-specific patterns observed across profiles suggest that the associations between primary emotional configurations, childhood adversity, attachment, and trauma-related psychopathology are not uniform across women and men. This observation is consistent with previous ANPS research documenting sex-related differences in specific primary emotional systems, particularly FEAR and PANIC (Fuchshuber et al., 2019; Orri et al., 2017; Sindermann et al., 2017). Importantly, the present findings extend this literature by suggesting that sex-related variation may also characterize the developmental and clinical correlates of person-centered affective configurations.

Overall, the findings support the utility of a person-centered approach for examining how primary emotional systems co-occur in relation to ACEs, attachment, and trauma-related psychopathology. Rather than indicating that particular profiles constitute affective endophenotypes or causal mechanisms, the results identify distinct patterns of co-occurring affective, developmental, interpersonal, and clinical characteristics. This perspective complements variable-centered approaches by considering the configuration of multiple primary emotional systems rather than their associations in isolation. Further longitudinal and multimethod research will be required to determine whether these affective configurations represent relatively stable individual differences, consequences of adverse experiences, or dynamic correlates of trauma-related psychopathology.

### 4.3. Limitations

Several limitations should be considered. First, the cross-sectional design precludes causal conclusions regarding the relationships among primary emotional systems, attachment, childhood adversity, and trauma-related psychopathology, highlighting the need for longitudinal studies. Further, despite the clear-cut associations found between distinct affective profiles, PTSD and CPTSD, attachment styles, and ACEs, the study design also prevents any generalization concerning their stability in time. In other words, the impermanence of mood and emotional responses is often a hallmark of psychopathology (Broome et al., 2015). Second, reliance on self-report measures may introduce recall bias and shared method variance, supporting the use of multimethod assessments. Third, the convenience sample of Portuguese adults and small subgroup sizes limit generalizability and warrant cautious interpretation. Fourth, LPA-derived profiles are probabilistic and require replication across samples. Fifth, participants were assigned to their most likely profile based on posterior probabilities, which does not fully account for individual-level classification uncertainty. Although the profile solution demonstrated good classification quality and robustness across additional analyses, some degree of uncertainty in profile assignment remains. Therefore, associations between profile membership and external variables should be interpreted with this consideration in mind. Future research could address this limitation by using three-step or BCH-type approaches that explicitly account for classification uncertainty. Sixth, the multinomial regression findings should be interpreted cautiously given the number of predictors and profile-specific contrasts examined. The absence of an adjustment for multiple comparisons represents a further limitation, as it may increase the risk of an inflated Type I error rate. Accordingly, these findings should be considered exploratory, particularly those involving *p*-values close to the conventional significance threshold of 0.05. A further limitation concerns the simultaneous inclusion of four mutually exclusive attachment indicators in the regression models, which may introduce multicollinearity and potentially affect the stability and precision of the estimated associations. Finally, unexamined factors, including social support, emotion regulation, coping, resilience, personality, and ongoing stressors, may all contribute differently to affective functioning with sex-specific influences. Therefore, future research should further investigate the biological, developmental, and sociocultural mechanisms that underly sex differences.

## 5. Conclusions

The present study extends AN personality research by demonstrating that primary emotional systems form distinct affective profiles that are differentially associated with childhood adversity, attachment, and trauma-related psychopathology. Four broadly comparable profiles emerged across women and men, although quantitative differences in specific emotional systems were observed. Profiles characterized by heightened negative affective activation or low affective activation were more strongly associated with insecure attachment, childhood adversity, and trauma-related psychopathology, whereas the Positive Affect profile was characterized by greater activation of SEEKING, CARE, and PLAY and lower negative affective activation.

Clinically, these findings suggest that profiling primary emotional systems may complement symptom-based assessment by capturing meaningful heterogeneity in affective and interpersonal functioning. Such profiles may inform case formulation and highlight the need to assess threat-related distress, emotional engagement, attachment insecurity, and interpersonal functioning. However, these implications remain hypothesis-generating, as the cross-sectional and self-report design precludes causal inference and does not support interpreting the profiles as diagnostic categories or affective endophenotypes. Longitudinal and multimethod research is needed to establish their stability and clinical utility.

## Figures and Tables

**Table 1:** Sample demographic characteristics.

Variables	*n*	%
**Sex**		
Female	579	63.1
Male	338	36.9
**Civil status**		
Single	507	55.3
Married or cohabitation	321	35.0
Divorced	77	8.4
Widowed	12	1.3
**Education**		
Less than nine years of education	17	1.9
Nine years of education	36	3.9
12 years of education	449	49.0
Higher education	415	45.2
**Professional status**		
Student	381	41.6
Employee	438	47.8
Unemployed	62	6.8
Retired	36	3.9
**Socioeconomic status**		
Low	76	8.3
Medium	643	70.1
Medium-high	182	19.8
High	16	1.7

**Table 2:** Fit indices for the latent profile analysis in the female subgroup.

Model	AIC	BIC	SSABIC	BLRT	*p*	Entropy	Smallest Group Proportion
2 classes	9595.74	9678.71	9618.29	289.25	0.001	0.70	40.6
3 classes	9458.36	9571.36	9489.22	151.38	0.001	0.72	15.4
4 classes	9372.20	9516.13	9411.36	100.16	0.001	0.73	15.4
5 classes	9351.07	9525.52	9398.54	35.13	0.001	0.69	13.6
6 classes	9327.38	9532.36	9383.15	37.70	0.001	0.71	6.6

**Table 3:** Fit indices for the latent profile analysis in the male subgroup.

Model	AIC	BIC	SSABIC	BLRT	*p*	Entropy	Smallest Group Proportion
2 classes	5491.09	5563.65	5503.38	155.21	0.001	0.61	48.2
3 classes	5399.26	5498.66	5416.18	105.75	0.001	0.66	25.7
4 classes	5354.93	5471.09	5376.41	58.33	0.001	0.76	9.8
5 classes	5328.70	5481.62	5354.73	50.23	0.001	0.77	7.4
6 classes	5301.52	5482.20	5333.11	30.18	0.001	0.78	5.3

**Table 4:** Measurement and structural invariance results by sex.

Model Comparison	LL (k) −2ΔLL (Δk)	BIC ΔBIC	Entropy ΔEntropy
M1. Unconstrained	−14,325 (102)	32,365	0.810
M2. Constrained	−14,696 (78)	31,070	0.778
M2 vs. M1	371 ***	−1295	0.032
M2a Partial constrained ^1^	−14,511 (81)	32,019	0.828
M2a vs. M1	186 ***	−346	0.018

*Note*. Models for the gender invariance of the latent profiles. LL = Log-likelihood; −2ΔLL = Log-likelihood ratio statistic; k = number of parameters; BIC = Bayesian Information Criterion; ^1^ free to vary: PANIC, CARE, and FEAR; *** *p* < 0.001.

**Table 5:** Tests of between-subject effects for sex.

Dependent Variables	Mean Square	Df	F	*p*	Partial Eta Squared	Female Group Mean (CI 95%)	Male Group Mean (CI 95%)
ANGER	509.23	1	27.41	0.001	0.03	17.44 (17.07–17.80)	15.89 (15.45–16.33)
FEAR	564.42	1	49.97	0.001	0.05	18.35 (18.08–18.63)	16.73 (16.38–17.08)
PANIC	1512.45	1	63.42	0.001	0.07	18.85 (18.46–19.25)	16.19 (15.66–16.72
SEEKING	0.10	1	0.01	0.93	0.00	21.87 (21.58–22.15)	21.84 (21.45–22.24)
CARE	31.12	1	3.81	0.05	0.01	15.29 (15.05–15.53)	14.91 (14.62–15.20)
PLAY	2.68	1	0.16	0.69	0.00	22.54 (22.19–22.87)	22.65 (22.23–23.07)

**Table 6:** Multinomial logistic regression predicting latent profile membership among female participants.

**Profile 1: Negative Affect**					
**Effect**	**−2 Log Likelihood**	**Chi-Square**	**df**	***p***	**OR (95% CI)**
Intercept	185.55	0.00	0		
Emotional abuse	186.06	0.72	1	0.40	0.74 (0.37; 1.49)
Physical abuse	187.41	1.81	1	0.18	0.57 (0.25; 1.30)
Sexual abuse	186.37	0.84	1	0.36	1.36 (0.71; 2.61)
Emotional neglect	190.46	4.19	1	0.04	2.01 (1.08; 3.35)
Physical neglect	188.11	2.59	1	0.11	1.64 (0.90; 3.00)
Secure	197.16	11.45	1	0.001	0.23 (0.10; 0.54)
Preoccupied	187.59	2.10	1	0.15	2.01 (0.78; 5.17)
Dismissing	197.41	11.63	1	0.001	4.44 (1.89; 7.48)
Fearful	202.31	51.11	1	0.001	5.52 (3.43; 8.90)
PTSD	190.35	9.92	1	0.003	2.58 (1.20; 6.53)
CPTSD	191.58	5.57	1	0.02	2.18 (1.33; 3.96)
**Profile 2: Low Affective Activation**					
**Effect**	**−2 Log Likelihood**	**Chi-Square**	**df**	***p***	**OR (95% CI)**
Intercept	190.37	0.00	0		
Emotional abuse	192.95	2.15	1	0.14	0.61 (0.32; 1.18)
Physical abuse	198.37	4.89	1	0.03	4.81 (1.20; 9.32)
Sexual abuse	192.29	1.24	1	0.41	1.92 (0.65; 5.66)
Emotional neglect	190.75	0.02	1	0.89	1.08 (0.36; 3.26)
Physical neglect	192.73	3.04	1	0.08	2.32 (0.90; 6.00)
Secure	192.33	1.26	1	0.26	1.47 (0.75; 2.89)
Preoccupied	190.85	0.06	1	0.81	1.13 (0.42; 3.07)
Dismissing	197.38	6.65	1	0.01	2.92 (1.30; 6.88)
Fearful	192.45	0.59	1	0.46	1.39 (0.59; 3.29)
PTSD	190.80	0.06	1	0.80	1.07 (0.63; 1.83)
CPTSD	193.05	4.55	1	0.03	2.34 (1.18; 5.71)
**Profile 3: Balanced Affective Functioning**					
**Effect**	**−2 Log Likelihood**	**Chi-Square**	**df**	***p***	**OR (95% CI)**
Intercept	259.31	0.00	0		
Emotional abuse	263.38	5.09	1	0.02	2.68 (1.14; 6.31)
Physical abuse	260.74	0.79	1	0.37	1.61 (0.57; 4.56)
Sexual abuse	260.97	1.32	1	0.25	1.60 (0.72; 3.56)
Emotional neglect	261.07	1.34	1	0.25	1.66 (0.70; 3.52)
Physical neglect	259.62	0.07	1	0.79	1.09 (0.56; 2.13)
Secure	259.41	0.01	1	0.94	1.03 (0.51; 2.06)
Preoccupied	264.53	20.17	1	0.001	4.11 (2.00; 8.42)
Dismissing	259.76	0.08	1	0.78	1.07 (0.66; 1.75)
Fearful	259.91	1.85		0.17	1.69 (0.79; 3.60)
PTSD	263.49	5.10	1	0.02	2.17 (1.11; 4.24)
CPTSD	260.28	0.35	1	0.55	1.43 (0.44; 4.65)

*Notes*. Reference group = Positive affect profile, n = 579. Nagelkerke R^2^ = 0.29.

**Table 7:** Multinomial logistic regression predicting latent profile membership among male participants.

**Profile 1: Negative Affect**					
**Effect**	**−2 Log Likelihood**	**Chi-Square**	**df**	***p***	**OR (95% CI)**
Intercept	92.48	0.00	0		
Emotional abuse	96.57	4.02	1	0.04	2.70 (1.11; 5.35)
Physical abuse	93.04	0.65	1	0.42	1.51 (0.53; 3.88)
Sexual abuse	92.35	0.15	1	0.70	1.22 (0.41; 3.58)
Emotional neglect	92.70	0.28	1	0.59	1.45 (0.47; 4.61)
Physical neglect	96.40	4.05	1	0.04	2.58 (1.06; 4.04)
Secure	97.48	7.44	1	0.006	0.20 (0.06; 0.63)
Preoccupied	94.56	0.14	1	0.83	1.09 (0.52; 2.87)
Dismissing	94.75	1.87	1	0.16	1.74 (0.89; 3.51)
Fearful	99.74	11.24	1	0.001	6.03 (2.11; 17.23)
PTSD	96.97	4.06	1	0.04	2.91 (1.04; 4.71)
CPTSD	97.08	5.19	1	0.02	3.70 (1.46; 6.64)
**Profile 2: Low Affective Activation**					
**Effect**	**−2 Log Likelihood**	**Chi-Square**	**df**	***p***	**OR (95% CI)**
Intercept	115.60	0.00	0		
Emotional abuse	117.09	2.03	1	0.15	2.11 (0.74; 5.90)
Physical abuse	125.12	8.64	1	0.003	5.70 (1.79; 9.20)
Sexual abuse	117.38	2.52	1	0.11	3.71 (0.74; 6.73)
Emotional neglect	124.09	5.80	1	0.02	5.03 (1.35; 8.71)
Physical neglect	115.71	0.06	1	0.81	1.12 (0.44; 3.03)
Secure	117.06	5.60	1	0.02	0.32 (0.12; 0.82)
Preoccupied	116.56	0.80	1	0.37	1.58 (0.63; 3.28)
Dismissing	122.68	4.07	1	0.04	1.87 (1.06; 3.04)
Fearful	123.06	4.89	1	0.03	2.29 (1.19; 5.58)
PTSD	116.83	1.70	1	0.19	1.53 (0.72; 3.25)
CPTSD	122.88	4.09	1	0.04	2.78 (1.14; 5.39)
**Profile 3: Balanced Affective Functioning**					
**Effect**	**−2 Log Likelihood**	**Chi-Square**	**df**	***p***	**OR (95% CI)**
Intercept	143.51	0.00	0		
Emotional abuse	145.76	2.15	1	0.14	2.12 (0.68; 4.23)
Physical abuse	143.66	0.11	1	0.74	1.28 (0.65; 3.67)
Sexual abuse	145.93	2.78	1	0.10	2.91 (0.84; 5.93)
Emotional neglect	146.14	3.73	1	0.06	1.78 (0.95; 3.88)
Physical neglect	145.03	0.93	1	0.34	1.62 (0.68; 3.58)
Secure	144.56	0.44	1	0.51	1.20 (0.70; 2.07)
Preoccupied	144.28	0.38	1	0.54	1.42 (0.47; 4.34)
Dismissing	144.87	0.68	1	0.41	1.58 (0.53; 4.67)
Fearful	145.12	0.97		0.33	1.63 (0.68; 3.25)
PTSD	144.68	0.58	1	0.49	1.48 (0.54; 4.03)
CPTSD	144.98	0.88	1	0.35	1.57 (0.78; 4.14)

*Notes*. Reference group = Positive affect profile, n = 338. Nagelkerke R^2^ = 0.28.

## Data Availability

The data presented in this study are available on request from the corresponding author due to data protection regulations.
